# The Prognostic Value of Serum Uric Acid in Hospitalized Patients with Acute Cerebral Infarction

**DOI:** 10.1155/2021/6103961

**Published:** 2021-09-30

**Authors:** Bangjian Liu, Yongchao Pan, Li Cao, Jiajun Yang

**Affiliations:** Department of Neurology, Shanghai Jiao Tong University Affiliated Sixth People's Hospital, Shanghai, China 200233

## Abstract

**Background:**

Previous studies reported that the level of serum uric acid (SUA) was an important risk factor for acute cerebral infarction (ACI). However, the prognostic value of SUA levels in hospitalized patients with ACI has not been fully elucidated. The aim of this study was to investigate whether the SUA level on admission was associated with subsequent mortality in hospitalized patients with ACI.

**Methods:**

The clinical data of ACI patients obtained from December 2017 to December 2019 were retrospectively reviewed. *χ*^2^ and Kaplan–Meier methods were used to compare the clinical differences and overall survival between patients with or without hyperuricemia, respectively. Univariate and multivariate analyses were used to identify independent prognoses.

**Results:**

In the total population, the in-hospital mortality of the hyperuricemia group was significantly higher than that of the normal uric acid group (*P* = 0.006). In the abnormal renal function group, the in-hospital mortality among the hyperuricemia group was significantly higher than the normal uric acid group (*P* = 0.002). However, there was no statistical difference of in-hospital mortality between the two groups in the normal renal function group (*P* = 0.321). Univariate and multivariate analyses showed that a previous history of diabetes (*P* = 0.018), hyperuricemia (*P* = 0.001), and National Institutes of Health Stroke Scale (NIHSS) score on admission (*P* ≤ 0.001) were independent factors for all samples. The hyperuricemia (*P* = 0.003) on admission were independent factors for patients with abnormal renal function.

**Conclusions:**

In ACI patients with abnormal renal function, hyperuricemia may be associated with higher in-hospital mortality than patients with normal uric acid, and hyperuricemia may be an independent associated factor for in-hospital death in the subgroup patients.

## 1. Introduction

Serum uric acid (SUA) is the final product of purine metabolism. SUA levels increase during the first hours after acute cerebral infarction (ACI) and decrease to baseline levels after a few days [1]. SUA levels may be related to age, sex, patients' physical characteristics, renal function, diet, and drug and alcohol intake [2]. The SUA levels depend on the quantities of production and excretion, and the decreased renal function is an important cause of hyperuricemia [3]. Increased levels of SUA (the product of purine metabolism) have an adverse effect on human tissues. Adhesion of uric acid crystals to the surface of epithelial cells can cause an inflammatory response [4]. The adverse effects of hyperuricemia also include increased serum levels of cytokines and tumor necrosis factor-*α* and increased local expression of chemokines, monocyte chemoattractant protein, and cyclooxygenase-2 in blood vessels [5]. It has also been reported that the increased SUA levels appear to be associated with endothelial dysfunction and are an early manifestation of vascular injury [6].

At present, there are many controversies about the relationship between hyperuricemia and the prognosis of ACI. Some studies have suggested that SUA has a protective effect on both the acute and convalescent phases of cerebral infarction [7]. SUA was a natural antioxidant, and increased levels have been associated with neuroprotective benefits in several neurodegenerative diseases and improvements in neuroimmunity [8]. Clinical studies have found that among ischemic stroke patients, those in the high SUA level group had better clinical outcomes [9, 10]. Several studies suggested that hyperuricemia had a protective effect on the prognosis of ACI [11, 12]. The neuroprotective role of SUA at the onset of cerebral infarction has also been demonstrated in animal models [13]. SUA reduced infarct volume in hyperglycemic mice, but it did not prevent vascular ICAM-1 upregulation and did not significantly reduce the number of neutrophils in the ischemic brain tissue [11]. However, some clinical studies have also found that hyperuricemia was associated with hypertension [14], metabolic syndrome [15], chronic kidney disease [16], cardiovascular disease [17], electrocardiography changes [18], cognitive dysfunction [19, 20], and nonvalvular atrial fibrillation [21]. Patients with hyperuricemia often had platelet dysfunction, coagulopathy, endothelial dysfunction, increased oxidative stress, thrombosis, and inflammation, and so on, suggesting that hyperuricemia was associated with a poor prognosis following acute vascular events. Some studies have also suggested that hyperuricemia is an independent risk factor for ACI, and the elevated SUA on admission predicts poor prognosis [22, 23]. Even Amaro et al. found that hyperuricemia was not significantly associated with ACI patient prognosis [24]. However, elevated SUA levels may simply represent altered purine metabolism or reduced SUA excretion in patients.

Thus far, the role of uric acid in acute cerebrovascular disease is still not well established. The aim of this study was to investigate the association between SUA and in-hospital mortality in patients with ACI and to assess the applicability of the levels of SUA in predicting in-hospital mortality in patients with ACI.

## 2. Materials and Methods

### 2.1. Subjects

A total of 275 patients with ACI who were diagnosed and treated at the Department of Neurology of the Sixth People's Hospital affiliated with Shanghai Jiaotong University were recruited from December 2017 to December 2019.

### 2.2. Inclusion Criteria and Exclusion Criteria

Inclusion criteria are as follows: (1) All enrolled patients had their first stroke, and there was no previous history of severe neurological deficits; the National Institutes of Health Stroke Scale (NIHSS) score ranged from 4 to 22 on admission; (2) the onset time was ≤48 hours, and cerebral infarction was diagnosed by computed tomography or magnetic resonance imaging within 48 hours; (3) the diagnosis of ACI was made in accordance with the Chinese Guidelines for the Diagnosis and Treatment of Acute Ischemic Stroke 2014; and (4) serum uric acid determination and clinical data were complete within 48 hours after the stroke. Exclusion criteria are as follows: (1) Patients with transient ischemic stroke, cerebral hemorrhage, or hemorrhagic transformation; (2) patients who had been given intravenous thrombolytics in the emergency room; (3) patients with a recent history of surgery; (4) patients with epilepsy; and (5) patients who had been taking xanthine oxidase inhibitors (allopurinol or febuxostat) before admission.

### 2.3. Methods

The demographic and clinical profile data of the study subjects were recorded, including age, sex, hospitalization days, NIHSS score on admission, SUA level, creatinine clearance values, and a diagnosis or the presence of heart failure, nonvalvular atrial fibrillation (NVAF), diabetes, hypertension, blood lipids, tumors, chronic obstructive pulmonary disease (COPD), and hospital-acquired infections. The data was independently entered into an Excel (Microsoft Corporation, Redmond, WA, USA) sheet by two members of the research group for analysis.

#### 2.3.1. Hypertension

Hypertension was defined in the presence of a history of increased blood pressure (BP) reported by the patient or the caregiver or an increased BP (≥140 mmHg for systolic BP and ≥90 mmHg for diastolic BP) reported by the general practitioner. We considered adequate to confirm this comorbidity or a previous diagnosis of hypertension or antihypertensive drugs use at the moment of the enrolment.

#### 2.3.2. Dyslipidemia

Dyslipidemia was considered when the patient or the caregiver reported a positive history for this condition. Alternatively, we considered diagnostic the chronic use of a statin or fibrates to treat this specific condition or fasting low-density lipoprotein cholesterol levels > 100 mg/dL during the hospitalization.

#### 2.3.3. Diabetes

Diabetes was diagnosed by a history of diabetes or the use of antidiabetic medications at the moment of the enrolment or by the presence of fasting plasma glucose ≥ 126 mg/dL or glycosylated hemoglobin > 6.5% during the hospitalization.

#### 2.3.4. Nonvalvular Atrial Fibrillation

We considered a diagnosis of NVAF when the patient or the caregiver reported a history of this condition or when the patient reported a use of anticoagulant or antiarrhythmic agents for this pathology. We also diagnosed this condition if we observed it in the ECGs performed in the emergency department or during the hospitalization.

#### 2.3.5. Cancer

A history of previous or active cancer was also investigated and added to the list of comorbidities in the database.

#### 2.3.6. Chronic Obstructive Pulmonary Disease

COPD was diagnosed when the patient or the caregiver reported this pathology at the first visit. We selected only patients with a diagnosis put by a lung specialist using spirometric data.

#### 2.3.7. Heart Failure

Heart failure was added to the list of comorbidities in the presence of at least one previous hospitalization for typical signs and symptoms and a subsequent clinical diagnosis of heart failure put by a cardiologist or an internal medicine specialist.

#### 2.3.8. Chronic Kidney Disease (CKD)

CKD was defined as the presence of a stable reduction in glomerular filtration rate (eGFR) eGFR < 90 mL/min/1.73 m^2^ for a period ≥ 90 days before hospital admission. We estimated eGFR according to chronic kidney disease epidemiology collaboration (CKD-EPI) equation in all subjects at their arrival in the emergency department.

#### 2.3.9. Hospital-Acquired Infections

From the admission in our department to the outcome (in-hospital death or end of hospitalization), we monitored and recorded the occurrence of hospital-acquired infections, defined as symptomatic urinary tract infections or pneumonia occurring at least 48 hours after hospital admission.

#### 2.3.10. National Institutes of Health Stroke Scale

All patients were marked by score of NIHSS in the emergency department to assess neurological impairment. The score ranged from 4 to 22 on admission.

### 2.4. Statistical Methods

The Statistical Package for the Social Sciences (SPSS) version 19.0 software (IBM Corporation, Armonk, NY, USA) was used for statistical analysis of the data. Categorical variables are expressed as percentages, and continuous variables are expressed as medians (IQRs). Comparisons for categorical baseline measurements were performed with Chi-square test, and continuous, not normally distributed baseline data were compared by Mann–Whitney *U* test. In-hospital death was the endpoint event, and the Kaplan–Meier method was applied to plot survival curves for the overall population, as well as for patients with normal renal function and patients with abnormal renal function. The log-rank test was used to analyze the risk factors that may affect patient prognosis, such as sex, age, hypertension, diabetes, heart failure, nonvalvular atrial fibrillation, hyperlipidemia, tumor, COPD, hospital-acquired infections, NIHSS score on admission, and SUA. Univariate analysis was performed for the short-term prognosis of cerebral infarction. The factors that exhibited statistical significance during the univariate analysis were included in the Cox proportional hazards regression model for the multivariate analysis of prognosis. *P* < 0.05 was considered statistically significant.

## 3. Results

### 3.1. Participant Selection

**Figure 1:**
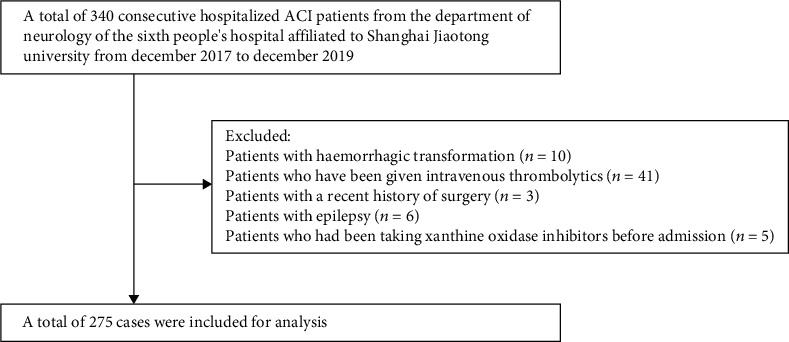
A flow chart for patients' screening.

Based on our strict screening criteria, a total of 275 patients were selected for the final data analysis. A total of 65 patients were excluded, including 10 patients with hemorrhagic transformation, 41 patients who had been given intravenous thrombolytics in the emergency room, three patients with a recent history of surgery, six patients with epilepsy, and five patients who had been taking xanthine oxidase inhibitors before admission (see Figure 1 for a flow chart).

### 3.2. Baseline Data and Demographic Characteristics

A total of 275 patients with ACI were included in this study, including 104 males and 171 females. The age of patients ranged from 55 to 95 years old. Overall median age was 81 years (IQR, 77–85 years), and 62.1% were females. Patients' SUA ranged from 209 to 600 *μ*mol/L with a median value of 367 *μ*mol/L. There was no significant difference in terms of sex, hypertension, atrial fibrillation, tumor, dyslipidemia, age, hospitalization days, creatinine clearance values, NIHSS scores on admission, as well as the proportion of patients with diabetes, heart failure, COPD, and CKD, and hospital-acquired infections between the two groups (Table 1).

### 3.3. Comparison of Survival Curves for Patients with ACI according to SUA Level

**Figure 2:**
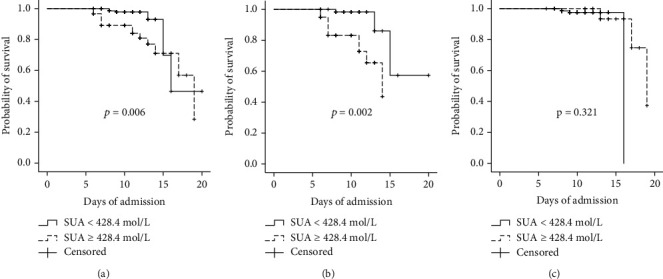
Kaplan–Meier survival curves for patients with ACI according to uric acid level. Cox proportional hazards model survival function in the complete sample (a), in the subgroup of patients with abnormal renal function (b), and with normal renal function (c), adopting a serum uric acid cut − off ≥ 428.4 *μ*mol/L.

The total population of patients satisfied with the condition of ACI, normal renal function, and abnormal renal function (eGFR < 90 mL/min/1.73 m^2^) were divided into groups according to the cut-off of SUA level of 428.4 *μ*mol/L. Then, the associated survival curves were analyzed. In the total population, the in-hospital mortality in the hyperuricemia group was significantly higher than that in the normal uric acid group (*P* = 0.006; Figure 2(a)). In the abnormal renal function group, the in-hospital mortality in the hyperuricemia group was significantly higher than that in the normal uric acid group (*P* = 0.002; Figure 2(b)). However, there was no significant difference in the in-hospital mortality between the two groups in the normal renal function group (*P* = 0.321; Figure 2(c)).

### 3.4. Univariate and Multivariate Analyses of Risk Factors for In-Hospital Death in Patients with ACI

According to 12 factors (sex, age, previous history of diabetes, previous history of hypertension, heart failure, atrial fibrillation, hyperlipidemia, tumor, COPD, hospital-acquired infections, SUA, and NIHSS score on admission) that may affect in-hospital mortality in patients with ACI, the stratified analysis of all included ACI patients were performed. Univariate analysis showed that age, a previous history of diabetes, atrial fibrillation, SUA level, and NIHSS score on admission were significantly associated with in-hospital mortality in patients with ACI (*P* < 0.05). Cox regression multivariate analysis was performed for those factors with statistical differences during the univariate analysis, and the results showed that a previous history of diabetes (*P* = 0.018), SUA level (*P* = 0.001), and NIHSS score on admission (*P* ≤ 0.001) were independent factors associated with in-hospital death in patients with ACI (Table 2).

### 3.5. Univariate and Multivariate Analyses of Risk Factors for In-Hospital Death in Cerebral Infarction Patients with Abnormal Renal Function

The univariate analysis showed that a previous history of diabetes, SUA levels, and NIHSS scores on admission were associated with in-hospital mortality among those with abnormal renal function, and the differences were statistically significant (*P* < 0.05). Cox regression multivariate analysis showed that SUA level (*P* = 0.003) on admission were independent factors for in-hospital death in ACI patients with abnormal renal function (Table 3).

### 3.6. Univariate and Multivariate Analyses of Risk Factors for In-Hospital Death in ACI Patients with Normal Renal Function

Univariate analysis showed that age and NIHSS score on admission were associated with in-hospital mortality, and the differences were statistically significant (*P* < 0.05). Cox multivariate regression analysis showed that NIHSS score on admission was an independent factor for in-hospital death in ACI patients with normal renal function (*P* = 0.049; Table 4).

## 4. Discussion

Stroke is one of the three most common causes of death, the fourth leading cause of productivity decline, and the major cause of disability worldwide. Findings from epidemiology show that stroke increases with age. The identification and correction of clinically relevant risk factors are the most direct way to reduce the incidence of stroke and to improve patient outcomes [25].

In the present study, Cox multivariate regression analysis showed that a previous history of diabetes (*P* = 0.018), SUA levels (*P* = 0.001), and NIHSS scores on admission (*P* ≤ 0.001) were independent factors for in-hospital death in patients with ACI. Our findings are consistent with other researchers who found an increase in stroke-related death rates among diabetic patients [26]. After controlling other variables, higher NIHSS scores were associated with increased mortality, which may be attributed to the fact that NIHSS is correlated with infarct volume in the acute phase of stroke and larger infarct volume is associated with more severe brain swelling [27]. This finding is consistent with the conclusion of the current study [28]. NIHSS scores have good predictive value for ACI patient prognosis.

In this study, 12 factors (sex, age, a previous history of diabetes, a previous history of hypertension, heart failure, nonvalvular atrial fibrillation, hyperlipidemia, tumor, COPD, hospital-acquired infections, SUA levels, and NIHSS score on admission) that may affect the in-hospital mortality of patients with ACI were analyzed via univariate analysis. We further performed Cox multivariate regression analysis for those factors that exhibited statistically significant differences during the univariate analysis and found that hyperuricemia was an independent risk factor for in-hospital mortality, but only for the patients with abnormal renal function group. These results suggested that hyperuricemia was associated with higher in-hospital mortality in the total population of patients and in the subgroup of patients with abnormal renal function. This was consistent with the findings of Falsetti et al. who demonstrated that hyperuricemia was found to be associated with poor outcomes in cerebral infarction patients with renal disease [2]. Patients with abnormal renal function might be associated with increased cerebral edema in the acute phase of ischemic stroke on account of poor blood pressure control [29], which affected the outcomes of cerebral infarction and increased mortality. In addition, hyperuricemia could be associated with aspirin resistance [29], which might directly affect the outcomes of ACI. This effect may be more pronounced in abnormal renal function patients because they have higher SUA levels and a higher risk of vascular disease than the general population.

Among patients with ACI, patients with hyperuricemia had a higher risk of in-hospital death [2]. Karagiannis et al. reported that elevated levels of SUA were independently associated with early death after ischemic stroke (median: 4 days after stroke; interquartile range: 2–7) [30]. However, patients with hyperuricemia often had more complications, as they were typically older in age [31] and may have poorly controlled hypertension [18], heart failure [31], CKD [32], metabolic syndrome [15], cardiovascular disease [17], diabetes [22], nonvalvular atrial fibrillation [21], and so on. These factors on their own might increase the risk of stroke and result in adverse outcomes following stroke. Further, the SUA level was found to be nonlinearly related to the degree of neurological impairment in patients with acute primary cerebral infarction [33]. When the SUA was <372 *μ*mol/L, it was negatively correlated with the degree of neurological impairment in patients with ACI. However, the protective effect of SUA disappeared when the SUA was ≥372 *μ*mol/L. According to our results, when the SUA was ≥428.4 *μ*mol/L, patients with abnormal renal function had increased in-hospital mortality. However, this phenomenon was not observed in patients with normal renal function.

Increased SUA levels were associated with a decline in renal function and poorly controlled blood pressure in hypertensive patients, which could partially explain the clinical outcomes we found in the present study. In fact, poorly controlled hypertension is a recognized risk factor for worse functional outcomes and an increased risk of death in ischemic stroke [34], which could directly affect ischemic stroke outcomes. Further, this effect could be stronger in patients affected by CKD.

At the same time, this study found that hyperuricemia was not a risk factor for in-hospital death in the group of ACI patients with normal renal function, confirming the findings by MiedemaI et al. that insofar as hyperuricemia was not found to be associated with stroke outcomes in people with a low prevalence of CKD [35].

Based on our findings, we can infer that SUA holds significant clinical significance for the prognosis of ACI, especially in patients with abnormal renal function. However, there is no clear evidence to support the treatment of hyperuricemia in patients at high risk of cardiovascular and cerebrovascular diseases, as treatment cannot significantly reduce vascular events. The literatures do not support the receiving treatment for hyperuricemia for asymptomatic patients at high risk of vascular disease [36]. Therefore, in ACI and other acute diseases, further randomized, double-blind, controlled trials are needed to verify the efficacy of xanthine oxidase inhibitor in hyperuricemia patients.

Of course, it is important to note that the present study has several limitations. For instance, it was small, mainly comprising patients with acute primary cerebral infarction, and all participants were Chinese. Further, the SUA levels may fluctuate significantly in the period of acute cerebral infarction; we did not explore changes of SUA levels after admission.

## 5. Conclusion

Hyperuricemia may be associated with higher in-hospital mortality among ischemic stroke patients. When the SUA level was >428.4 *μ*mol/L, it was found that the SUA levels were positively associated with in-hospital mortality in ACI patients.

In ACI patients with abnormal renal function, hyperuricemia may be associated with higher in-hospital mortality than patients with normal uric acid, and hyperuricemia may be an independent prognostic factor in the subgroup patients.

## Figures and Tables

**Table 1:** The baseline characteristics of the patients grouped by SUA levels (cut − off ≥ 428.4 *μ*mol/L).

Characteristics	Overall (*n* = 275)	SUA < 428.4 *μ*mol/L (*n* = 216)	SUA ≥ 428.4 *μ*mol/L (*n* = 59)	*P*
Age, median (IQR) (years)	81 (77, 85)	80 (77, 84)	83 (77, 88)	0.091
Sex (*n*, % of females)	171 (62.1)	132 (61.1)	39 (66.1)	0.484
Days of admission, median (IQR) (days)	10 (9, 12)	10 (8, 11)	11 (10, 13)	0.178
Creatinine clearance, median (IQR) (mL/min)	78 (37, 109)	83 (52, 113)	48 (26, 98)	0.064
NIHSS, median (IQR)	11 (8, 14)	10 (8, 13)	13 (10, 17)	0.059
Hypertension (*n*, %)	146 (53.1)	116 (53.7)	30 (50.8)	0.697
Diabetes (*n*, %)	62 (22.5)	51 (23.6)	11 (18.6)	0.418
Heart failure (*n*, %)	103 (37.5)	76 (35.2)	27 (45.8)	0.137
Atrial fibrillation (*n*, %)	46 (16.7)	32 (14.8)	14 (23.7)	0.104
Dyslipidemia (*n*, %)	41 (14.9)	32 (14.8)	9 (15.3)	0.933
Cancer (*n*, %)	15 (5.5)	12 (5.6)	3 (5.1)	0.888
COPD (*n*, %)	43 (15.6)	31 (14.4)	12 (20.3)	0.262
CKD (*n*, %)	100 (36.4)	74 (34.3)	26 (44.1)	0.165
Hospital-acquired infections (*n*, %)	45 (16.4)	35 (16.2)	10 (16.9)	0.891

Abbreviations: IQR: interquartile range; SUA: serum uric acid; NIHSS: National Institutes of Health Stroke Scale; COPD: chronic obstructive pulmonary disease; CKD: chronic kidney disease. The results are presented as *n* (%) or median (IQRs).

**Table 2:** Univariate and multivariate Cox proportional hazard regression analyses in the overall sample.

Factor	Univariate analysis		Multivariate analysis	
	HR (95% CI)	*P*	HR (95% CI)	*P*
Sex	1.586 (0.666, 3.774)	0.297		
Age	1.164 (1.051, 1.290)	0.004	1.031 (0.941, 1.130)	0.517
Hypertension	1.199 (0.500, 2.874)	0.685		
Diabetes	2.928 (1.228, 6.985)	0.015	3.182 (1.217, 8.323)	0.018
Heart failure	2.399 (0.956, 6.018)	0.062		
Atrial fibrillation	3.700 (1.410, 9.707)	0.008	1.407 (0.455, 4.355)	0.554
Dyslipidemia	1.359 (0.523, 3.527)	0.529		
Cancer	1.104 (0.145, 8.398)	0.924		
COPD	2.430 (0.977, 6.040)	0.056		
Hospital-acquired infections	1.845 (0.700, 4.862)	0.215		
NIHSS	1.394 (1.208, 1.608)	≤0.001	1.349 (1.159, 1.571)	≤0.001
SUA (cut − off ≥ 428.4 *μ*mol/L)	3.871 (1.608, 9.320)	0.003	6.589 (2.210, 19.645)	0.001

Abbreviations: HR: hazard ratio; CI: confidence interval; COPD: chronic obstructive pulmonary disease; NIHSS: National Institutes of Health Stroke Scale; SUA: serum uric acid.

**Table 3:** Univariate and multivariate Cox proportional hazard regression analyses in the subpopulation with abnormal renal function (eGFR < 90 mL/min/1.73 m^2^).

Factor	Univariate analysis		Multivariate analysis	
	HR (95% CI)	*P*	HR (95% CI)	*P*
Sex	1.151 (0.399, 3.322)	0.795		
Age	1.100 (0.960, 1.261)	0.168		
Hypertension	1.105 (0.392, 3.115)	0.850		
Diabetes	3.128 (1.068, 9.165)	0.038	2.977 (0.973, 9.105)	0.056
Heart failure	2.817 (0.867, 9.148)	0.085		
Atrial fibrillation	3.180 (0.966, 10.465)	0.057		
Dyslipidemia	1.534 (0.425, 5.533)	0.513		
Cancer	0.897 (0.116, 6.953)	0.917		
COPD	2.147 (0.737, 6.252)	0.161		
Hospital-acquired infections	1.718 (0.525, 5.627)	0.371		
NIHSS	1.340 (1.107, 1.623)	0.003	1.369 (1.119, 1.674)	0.061
SUA (cut − off ≥ 428.4 *μ*mol/L)	5.480 (1.519, 19.767)	0.009	8.577 (2.115, 34.784)	0.003

Abbreviations: HR: hazard ratio; CI: confidence interval; COPD: chronic obstructive pulmonary disease; NIHSS: National Institutes of Health Stroke Scale; SUA: serum uric acid.

**Table 4:** Univariate and multivariate Cox proportional hazard regression analyses in the subpopulation with normal renal function.

Factor	Univariable analysis		Multivariable analysis	
	HR (95% CI)	*P*	HR (95% CI)	*P*
Sex	7.687 (0.829, 71.316)	0.073		
Age	1.213 (1.005, 1.465)	0.044	1.105 (0.817, 1.494)	0.516
Hypertension	1.235 (0.223, 6.860)	0.809		
Diabetes	1.452 (0.258, 8.181)	0.672		
Heart failure	2.287 (0.382, 13.704)	0.365		
Atrial fibrillation	5.247 (0.703, 39.147)	0.106		
Dyslipidemia	1.207 (0.213, 6.852)	0.832		
Cancer	1.047 (0.026, 10.315)	0.824		
COPD	1.555 (0.214, 11.316)	0.663		
Hospital-acquired infections	1.106 (0.166, 7.354)	0.917		
NIHSS	1.488 (1.136, 1.949)	0.004	1.375 (1.000, 1.891)	0.049
SUA (cut − off ≥ 428.4 *μ*mol/L)	1.217 (0.207, 7.173)	0.828		

Abbreviations: HR: hazard ratio; CI: confidence interval; COPD: chronic obstructive pulmonary disease; NIHSS: National Institutes of Health Stroke Scale; SUA: serum uric acid.

## Data Availability

The data used to support the findings of this study are available from the corresponding author upon request.

## References

[1] Yang Y., Zhang Y., Li Y. (2018). U-shaped relationship between functional outcome and serum uric acid in ischemic stroke. *Cellular Physiology and Biochemistry : International Journal of Experimental Cellular Physiology, Biochemistry, and Pharmacology*.

[2] Falsetti L., Capeci W., Tarquinio N. (2017). Serum uric acid, kidney function and acute ischemic stroke outcomes in elderly patients: a single-cohort, perspective study. *Neurology international*.

[3] Srivastava A., Kaze A. D., McMullan C. J., Isakova T., Waikar S. S. (2018). Uric acid and the risks of kidney failure and death in individuals with CKD. *American journal of kidney diseases : the official journal of the National Kidney Foundation*.

[4] Yang X., Gu J., Lv H. (2019). Uric acid induced inflammatory responses in endothelial cells via up- regulating(pro)renin receptor. *Biomedicine & pharmacotherapy = Biomedecine & pharmacotherapie*.

[5] Kang D. H., Park S. K., Lee I. K., Johnson R. J. (2005). Uric acid-induced C-reactive protein expression: implication on cell proliferation and nitric oxide production of human vascular cells. *Journal of the American Society of Nephrology : JASN*.

[6] Zhang P., Wang H., Chen X. H., Liang W. Y., Liu W. W., Liu M. L. (2020). Effect of low-dose aspirin on serum uric acid levels in Chinese individuals over 60: subanalysis of a multicentre randomized clinical trial. *European Review for Medical and Pharmacological Sciences*.

[7] Waring W. S. (2002). Uric acid: an important antioxidant in acute ischaemic stroke. *QJM : Monthly Journal of the Association of Physicians*.

[8] Roumeliotis S., Roumeliotis A., Dounousi E., Eleftheriadis T., Liakopoulos V. (2019). Dietary antioxidant supplements and uric acid in chronic kidney disease: a review. *Nutrients*.

[9] Arévalo-Lorido J. C., Carretero-Gómez J., Robles N. R. (2018). Serum uric acid levels and outcome during admission in acute ischaemic stroke, depending on renal function. *The International Journal of Neuroscience*.

[10] Pan B. L., Wu L., Pan L. (2018). Up-regulation of microRNA-340 promotes osteosarcoma cell apoptosis while suppressing proliferation, migration, and invasion by inactivating the CTNNB1-mediated Notch signaling pathway. *Bioscience reports*.

[11] Justicia C., Salas-Perdomo A., Pérez-de-Puig I. (2017). Uric acid is protective after cerebral ischemia/reperfusion in hyperglycemic mice. *Translational Stroke Research*.

[12] Wang Y. F., Li J. X., Sun X. S., Lai R., Sheng W. L. (2018). High serum uric acid levels are a protective factor against unfavourable neurological functional outcome in patients with ischaemic stroke. *The Journal of International Medical Research*.

[13] Dhanesha N., Vázquez-Rosa E., Cintrón-Pérez C. J. (2018). Treatment with uric acid reduces infarct and improves neurologic function in female mice after transient cerebral ischemia. *Journal of Stroke and Cerebrovascular Diseases : The Official Journal of National Stroke Association*.

[14] Stewart D. J., Langlois V., Noone D. (2019). Hyperuricemia and hypertension: links and risks. *Integrated blood pressure control*.

[15] Fu S., Luo L., Ye P., Xiao W. (2015). Epidemiological associations between hyperuricemia and cardiometabolic risk factors: a comprehensive study from Chinese community. *BMC cardiovascular disorders*.

[16] Prasad Sah O. S., Qing Y. X. (2015). Associations between hyperuricemia and chronic kidney disease: a review. *Nephro-urology monthly*.

[17] Wu J., Qiu L., Cheng X. Q. (2017). Hyperuricemia and clustering of cardiovascular risk factors in the Chinese adult population. *Scientific reports*.

[18] Cicero A. F., Rosticci M., Tocci G. (2015). Serum uric acid and other short-term predictors of electrocardiographic alterations in the Brisighella Heart Study cohort. *European Journal of Internal Medicine*.

[19] Zhang J., Tang L., Hu J., Wang Y., Xu Y. (2020). Uric acid is associated with cognitive impairment in the elderly patients receiving maintenance hemodialysis-a two-center study. *Brain Behav*.

[20] Xu Y., Wang Q., Cui R., Lu K., Liu Y., Zhao Y. (2017). Uric acid is associated with vascular dementia in Chinese population. *Brain Behav*.

[21] Maharani N., Kuwabara M., Hisatome I. (2016). Hyperuricemia and atrial fibrillation. *International Heart Journal*.

[22] Wang P., Li X., He C. (2019). Hyperuricemia and prognosis of acute ischemic stroke in diabetic patients. *Neurological Research*.

[23] Tariq M. A., Shamim S. A., Rana K. F., Saeed A., Malik B. H. (2019). Serum uric acid - risk factor for acute ischemic stroke and poor outcomes. *Cureus*.

[24] Amaro S., Urra X., Gómez-Choco M. (2011). Uric acid levels are relevant in patients with stroke treated with thrombolysis. *Stroke*.

[25] Xu Y., Wang K., Wang Q., Ma Y., Liu X. (2021). The antioxidant enzyme PON1: a potential prognostic predictor of acute ischemic stroke. *Oxidative medicine and cellular longevity*.

[26] Johnston K. C., Bruno A., Pauls Q. (2019). Intensive vs standard treatment of hyperglycemia and functional outcome in patients with acute ischemic stroke: the SHINE randomized clinical trial. *JAMA*.

[27] Huang Z. X., Gu H. Q., Yang X., Wang C. J., Wang Y. J., Li Z. X. (2021). Risk factors for in-hospital mortality among acute ischemic stroke patients in China: a nationwide prospective study. *Neurological research*.

[28] Zhao X. J., Li Q. X., Liu T. J. (2018). Predictive values of CSS and NIHSS in the prognosis of patients with acute cerebral infarction: a comparative analysis. *Medicine*.

[29] Yildiz B. S., Ozkan E., Esin F. (2016). Does high serum uric acid level cause aspirin resistance?. *Blood coagulation & fibrinolysis : an international journal in haemostasis and thrombosis*.

[30] Karagiannis A., Mikhailidis D. P., Tziomalos K. (2007). Serum uric acid as an independent predictor of early death after acute stroke. *Circulation journal : official journal of the Japanese Circulation Society*.

[31] Roy-O'Reilly M., McCullough L. D. (2018). Age and sex are critical factors in ischemic stroke pathology. *Endocrinology*.

[32] Wang Z., Lin Y., Liu Y. (2016). Serum uric acid levels and outcomes after acute ischemic stroke. *Molecular Neurobiology*.

[33] Wang R., Zhong Y., Zhou Q., Xu P. (2020). Relationship between uric acid level and severity of acute primary cerebral infarction: a cross-sectional study. *BioMed research international*.

[34] Leonardi-Bee J., Bath P. M., Phillips S. J., Sandercock P. A., I S T C Group (2002). Blood pressure and clinical outcomes in the International Stroke Trial. *Stroke*.

[35] Miedema I., Uyttenboogaart M., Koch M., Kremer B., de Keyser J., Luijckx G. J. (2012). Lack of association between serum uric acid levels and outcome in acute ischemic stroke. *Journal of the Neurological Sciences*.

[36] REPOSI Investigators, Pasina L., Brucato A. L. (2014). Inappropriate prescription of allopurinol and febuxostat and risk of adverse events in the elderly: results from the REPOSI registry. *European Journal of Clinical Pharmacology*.

